# Fellow-Workers and the Roll of Honour

**Published:** 1915-12-11

**Authors:** 


### ROUND THE WORLD.

[The Editor invites Early Information and Contributions Marked "Fellow-Workers."]

### Fellow-Workers and the Roll of Honour.

Lieutenant Maurice Mackenzie, R.A.M.C., attached 2nd Battalion Royal Irish Rifles, is reported killed in action in France. Lieutenant Mackenzie was educated at Elstree and Repton, and received his medical training at the London Hospital, at which institution he had held a resident appointment. He obtained his M.R.C.S., L.R.C.P. qualifications three years ago, and was gazetted lieutenant in January this year. He was the youngest son of the late Sir Stephen Mackenzie.

Captain Francis Shingleton Smith, B.A. (Cantab.), M.R.C.S., L.R.C.P., I.M.S., attached 120th Infantry, who was killed in the fighting near Baghdad at the beginning of the fourth week of November, entered the Indian Medical Service in 1907, and was appointed to the rank of captain in 1910. He obtained the Conjoint M.R.C.S., L.R.C.P. qualifications in 1906, and for some time held a resident appointment at the Weston-super-Mare Hospital.

Captain D. Arthur, I.M.S., attached 7th Rajputs, obtained his M.B. degree at Glasgow University in 1907; two years previously he became a Bachelor of Science of the same University. He was wounded in the fighting near Baghdad, November 22 to 24.

Captain R. Knowles, I.M.S., attached 110th Mahrattas, who was wounded during the same engagements, obtained the Conjoint M.R.C.S., L.R.C.P. qualifications in 1907.

Lieutenant Maneckji Burjorji Patel, I.M.S., attached 7th Rajputs, who is reported wounded in the list of casualties in the Persian Gulf published on December 3, received his medical education at Edinburgh, and obtained the L.R.C.P., L.R.C.S. in 1908.

Captain W. C. Spackman, I.M.S., attached 48th Pioneers, whose name appears among the wounded in the same list, formerly resided at Wolverhampton. He obtained the M.R.C.S., L.R.C.P. in 1913.

Captain G. Tate, L.M.S.S.A., I.M.S., attached 104th Rifles, was also wounded in the engagements near Baghdad, November 22 to 24.

Surgeon-General Sir David Bruce, C.B., F.R.S., to whom the mathematics and physical section of the Royal Dutch Academy of Science has awarded the Leeuwenhoek Gold Medal for 1915, entered the Royal Army Medical Corps in 1883. He served in Malta and Egypt from 1884 to 1889, and in the latter year was appointed Assistant Professor of Pathology at the Army Medical School, Netley, which post he held until 1894. From 1894 to 1901 he served in South Africa, and was in Ladysmith during the siege, being specially promoted and decorated for his services on that occasion. He was a member of the Army Medical Advisory Board from 1902 to 1911, and editor of the *R.A.M.C. Journal* from 1904 to 1908. In 1903 and 1908 he went to Uganda as director of the Royal Society's Commission for the Investigation of Sleeping Sickness. He also went to Malta in 1904-5-6 as director of the Commission for the Investigation of Mediterranean Fever, and subsequently to Nyassaland as director of the Royal Society's Commission to investigate the connection between the wild animals and human and stock diseases. The distinction he has just received at the hands of the Dutch Academy is one of many honours that have been conferred upon him by British and foreign societies. The Leeuwenhoek medal was founded in 1875, and is awarded every ten years. It is one of the highest scientific rewards in Holland, and one of its most illustrious recipients was the late Professor Pasteur.

Lieutenant-Colonel H. O. B. Browne-Mason, R.A.M.C., whose name appears in the list published on December 7 of those wounded in the Persian Gulf, was a major in the Army Medical Service when war broke out, and has since been promoted. He is an old St. Mary's man, and obtained the M.R.C.S., L.R.C.P. in 1896.

Professor W. St. Clair Symmers, who has been requested by the War Office to go out to the Dardanelles as a consulting surgeon to the British Forces, is Professor of Pathology at Queen's University, Belfast. Previous to his acceptance of this post he had been pathologist to the Birmingham General Hospital and Professor of Bacteriology and Pathology at the School of Medicine, Cairo. His contributions to medical literature have been many and varied. In announcing Professor Symmers' impending departure, the Vice-Chancellor of the University stated that the Professor's special duty would be to aid in the treatment of wounded men by means of certain methods on which he had been engaged for some time, and which had now been brought to completion with most remarkable results.

Dr. T. S. Kirk, who will accompany Professor Symmers, holds various hospital appointments in Belfast. He is senior surgeon to the Belfast Children's Hospital, surgeon to the Forster Green Hospital for Consumption, and surgeon to the Royal Victoria Hospital.

Temporary-Lieutenant A. Noel Minns, 39th Field Ambulance, R.A.M.C., who, as recorded in The Hospital of November 6, gained his D.S.O. for gallant conduct on the Gallipoli Peninsula, contributes an extremely interesting letter to the current issue of *Guy's Hospital Gazette*. Writing from Suvla Bay, he says: "We have been out here for over three months, and out of ten M.O.s who first came out with the ambulance, only myself and two others are left, and I am running the show, whilst there are considerably less than half the crowd that we came out with, and of those that are left very few are in a fit condition to carry a stretcher more than a mile. We have taken part in every scrap of any importance since we arrived, and the whole time we have been here we have never been out of range of shell fire or even bullets." "However," adds Lieutenant Minns, "I am still running about as full of life as ever." He writes of amputations in a shed by candle-light, interrupted by the passage of a shell, and of lying flat on the ground dressing men while bullets whistled overhead. As Lieutenant Minns suggests, such exciting experiences certainly would be a good cure for those people who are too bored and *blasé* to take any interest in anything.

